# Acute esophageal necrosis with esophagus perforation treated by thoracoscopic subtotal esophagectomy and reconstructive surgery on a secondary esophageal stricture: a case report

**DOI:** 10.1186/s40792-019-0636-3

**Published:** 2019-05-08

**Authors:** Ryujiro Akaishi, Yusuke Taniyama, Tadashi Sakurai, Hiroshi Okamoto, Chiaki Sato, Michiaki Unno, Takashi Kamei

**Affiliations:** Department of Surgery, Tohoku University Graduate School of Medicine, 1-1 Seiryo-machi, Aoba-ku, Sendai, Miyagi 980-8574 Japan

**Keywords:** Acute esophageal necrosis, Esophageal perforation, Esophageal stricture, Surgical intervention

## Abstract

**Background:**

Acute esophageal necrosis is defined as necrosis of the esophageal mucosa causing diffuse black pigmentation of the esophagus, the so-called black esophagus from its endoscopic findings. The prevalence is only 0.001~0.2%, while its mortality rate is up to 32%. However, most of the cases are fatal by comorbidities.

**Case presentation:**

A 67-year-old female with diabetes mellitus was transported to the emergency room with hematemesis and disordered consciousness. She had suffered from nausea and epigastralgia for 2 days. The patient’s general status was shock evidenced by vital signs, and she did not respond to rehydration. After intubation, emergency endoscopic examination revealed black pigmentation of the esophageal mucosa, and the condition was diagnosed as acute esophageal necrosis. Antibiotics and plasmapheresis had been started, and the patient gradually stabilized. One week after the admission, esophagus perforation was suspected from the significant increase of the right pleural effusion and free air at the esophagus wall and the mediastinum on CT scan. Emergency thoracoscopy revealed an edematous esophagus which was colored black. Esophagectomy with esophagostomy and enterostomy was performed.

On resected specimen, mucosal necrosis was found only on the squamous epithelium, with three perforating areas in the middle to lower thoracic esophagus. No signs of inflammation or ischemia were found on the gastric mucosa of the esophagogastric junction. After the operation, the patient recovered generally well, except for the severe stenosis of the cervical esophagus. Cervical esophagectomy, tracheotomy, and anterior thoracic route reconstruction with free jejunum interposition and gastric tube were performed 9 months after the first surgery. No postoperative complications occurred; on the 37th day after the operation, the patient was eating well and was transferred to continue swallowing rehabilitation.

**Conclusion:**

It is important to detect the esophagus perforation and mediastinitis early and thereby not to miss the chance of surgical intervention to save the patient’s life. Surgery should be minimized, and reconstruction should be considered next.

If the cervical esophagus is also affected, reconstruction surgery should be performed by removing cervical esophagus and anastomosing it to the hypopharynx using a gastric tube and free jejunum interposition as needed.

## Background

Acute esophageal necrosis (AEN), also called black esophagus, is characterized by the endoscopic finding of black friable mucosa, usually in the distal two thirds of the esophagus [1]. The etiology of AEN is assumed to be multifactorial and usually results from a combination of tissue hypoperfusion, impaired local defense barriers, and gastric acid reflux affecting the vulnerable esophageal mucosa [2–4]. AEN is a rare disease, and its prevalence ranges from 0.001 to 0.2% [5]. The overall mortality of AEN is reported to be 31.8%, while most cases are fatal due to comorbidities and complications [2, 5, 6]. Mortality specific to AEN is much lower, at approximately 6% [2].

Esophageal perforation is a complication in the acute phase of AEN, and its incidence is 6.8% [2]. Moreover, esophageal perforation is a lethal complication that requires surgical intervention [2]. Even after recovery from the acute phase, esophageal stricture might develop in 10.2~20% of AEN cases [2, 7]. AEN-induced esophageal stricture is often refractory to conservative therapy, although very few cases of surgical intervention have been reported [7–9].

Herein, we report a rare case of AEN with surgical intervention to esophageal perforation by performing a thoracoscopic subtotal esophagectomy and a successful reconstruction surgery for the stricture of the residual cervical esophagus.

## Case presentation

**Fig. 1:**
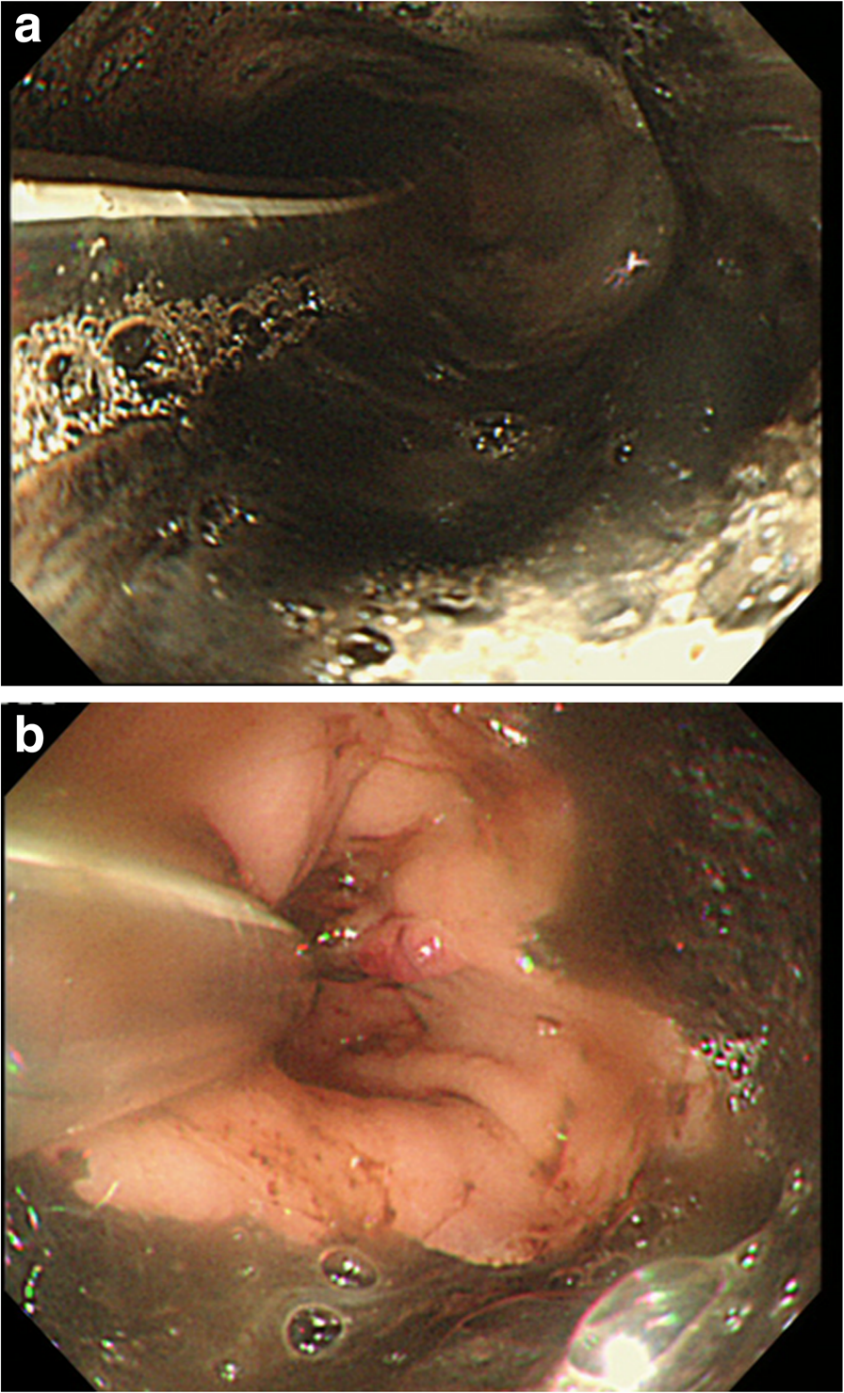
Emergency endoscopic findings. **a** Black discoloration of the mucosa of the entire esophagus. **b** The border of the lesion at the esophagogastric junction

**Fig. 2:**
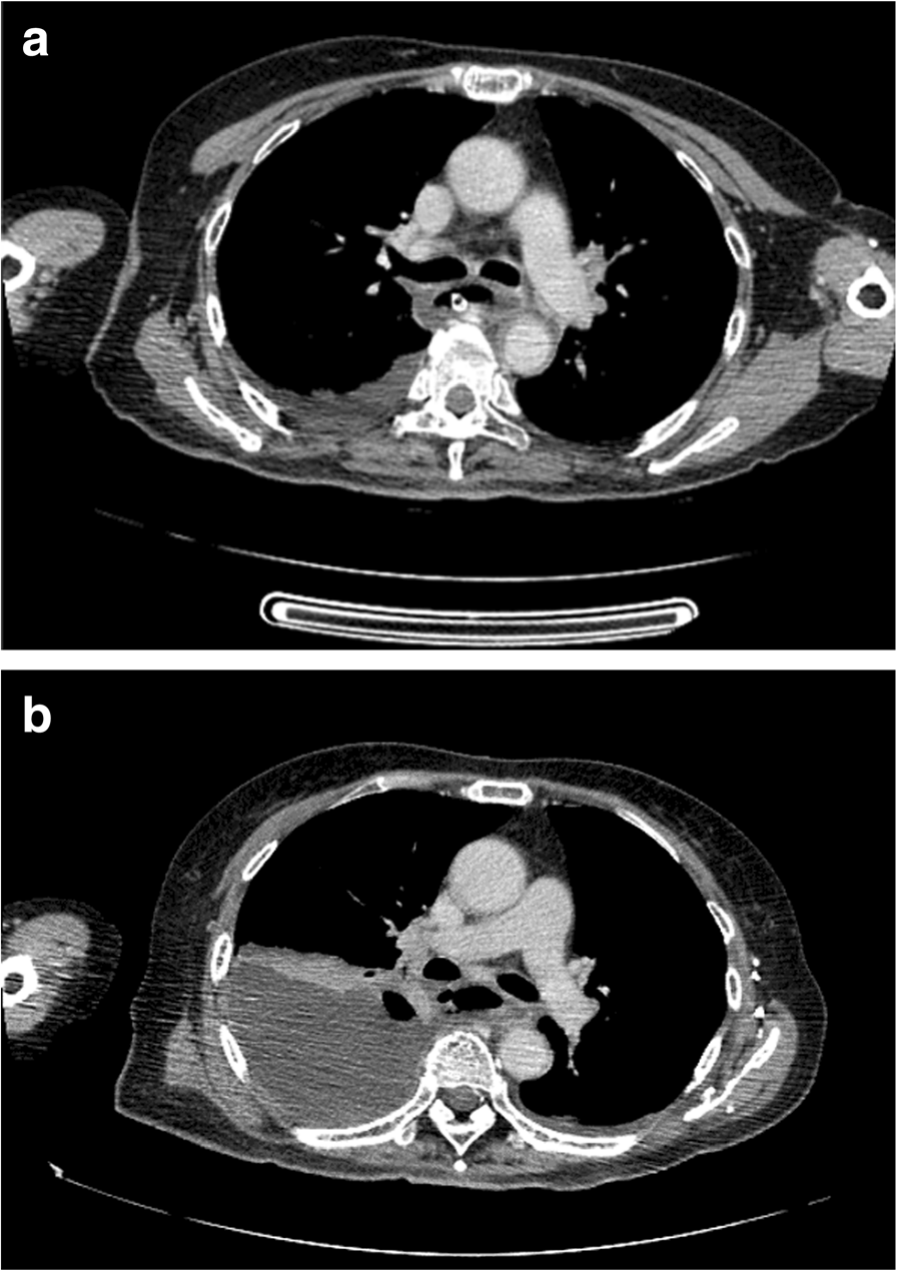
CT scan on admission day and on the 7th day after admission. **a** Circumferential edematous thickening of the esophageal wall with slight pleural effusion. **b** CT scan showed a significant increase of the right pleural effusion and free air in the mediastinum

A 67-year-old female with a medical history of poorly controlled diabetes mellitus transported to the emergency room with hematemesis and disordered consciousness. She had suffered from nausea and epigastralgia for 2 days. The patient’s general status was shock evidenced by vital signs, and she did not respond to rehydration. Laboratory findings showed blood glucose of 470 mg/dL; arterial blood gas with a pH of 7.2, PCO_2_ of 25.2 mmHg, HCO_3_ of 9.9 mEq/L, and PO_2_ of 169 mmHg with an anion gap of 24.3 mEq/L; and positive urinary ketones and glucose. These findings were consistent with the diagnosis of diabetic ketoacidosis. After intubation, emergency endoscopy revealed black pigmentation in the entire esophageal mucosa (Fig. 1a, b). A CT scan revealed the circumferential edematous thickening of the esophageal wall with slight pleural effusion (Fig. 2a). However, no obvious sign of perforation, such as free air in the mediastinum, was observed. AEN was diagnosed from these findings, and antibiotics, glycemic control, proton pump inhibitor (PPI), and plasmapheresis were started. The patient gradually stabilized and was extubated on day 5. However, she still had a fever, and the chest X-ray revealed the accumulation of pleural effusion. On day 7, esophageal perforation was suspected from the significant increase of the right pleural effusion and free air in the mediastinum on CT scan (Fig. 2).

**Fig. 3:**
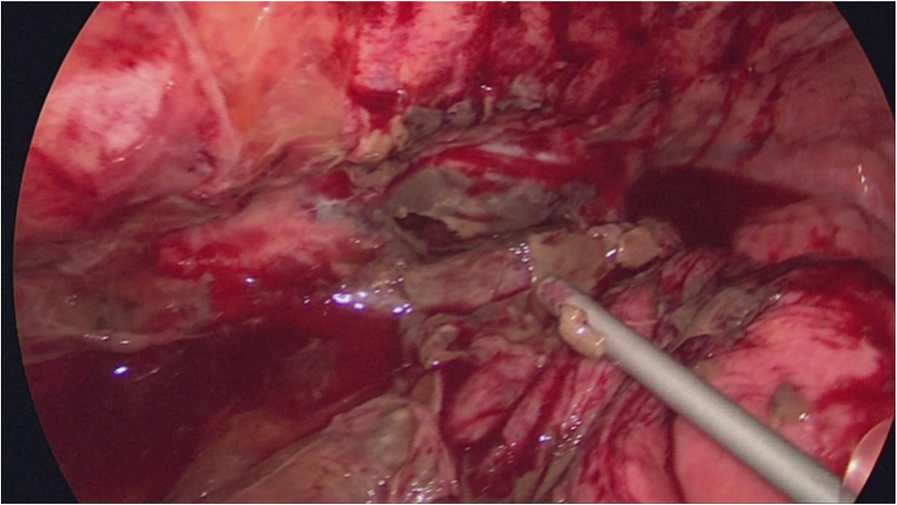
Findings of emergency thoracoscopy. Emergency thoracoscopy revealed necrosis and perforation of the esophagus

**Fig. 4:**
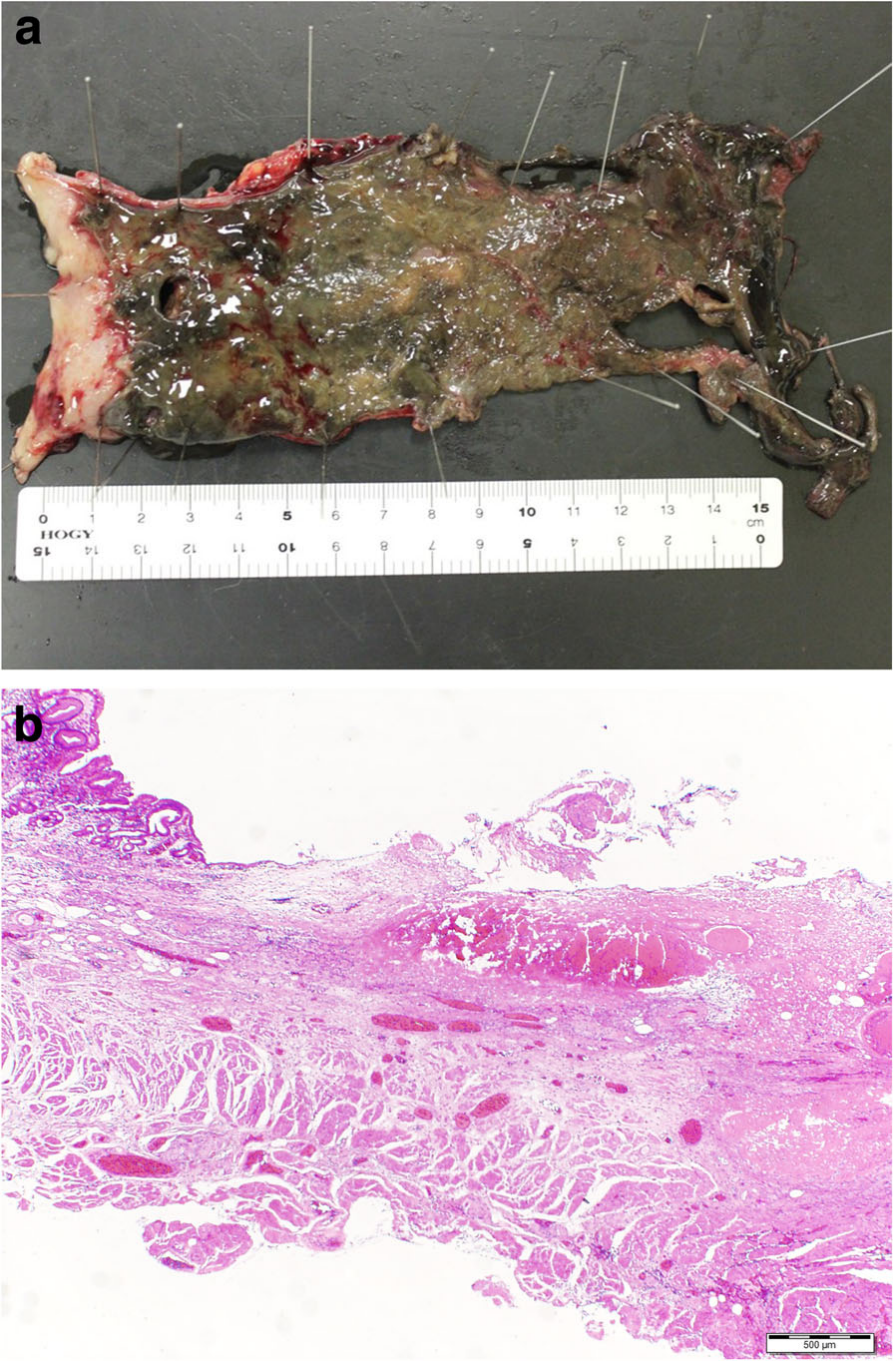
Findings of surgical and pathologic specimen (× 20 HE). **a** Black pigmented esophageal mucosa and perforation of the esophagus. No discoloration in the gastric mucosa. **b** Necrosis and deciduation of the esophageal mucosa. Gastric mucosa remained intact

Emergency thoracoscopy was performed in the left semiprone position with differential lung ventilation and artificial pneumothorax. Significant pleural effusion with pus and perforation of the esophagus was observed (Fig. 3). We resected the transmural necrotic thoracic esophagus and placed drainage tubes at the anterior side of the thoracic cavity, at the posterior mediastinum, and above the diaphragm. After transitioning the patient to the supine position, the esophagus was resected at the cardia of the stomach and the esophageal hiatus was closed. Then, an enterostomy tube was inserted from the upper jejunum, and the drainage tube was placed at the stump of the stomach. Finally, we constructed an esophagostomy with the residual cervical esophagus in the supraclavicular area. The total duration of the operation was 6 h and 33 min. On the resected specimen, mucosal necrosis was found only on the squamous epithelium, with a perforated area in the middle to lower thoracic esophagus (Fig. 4a). Pathological findings revealed necrosis of the esophageal mucosa, while no signs of inflammation or ischemia were found on the gastric mucosa of the esophagogastric junction (Fig. 4b).

**Fig. 5:**
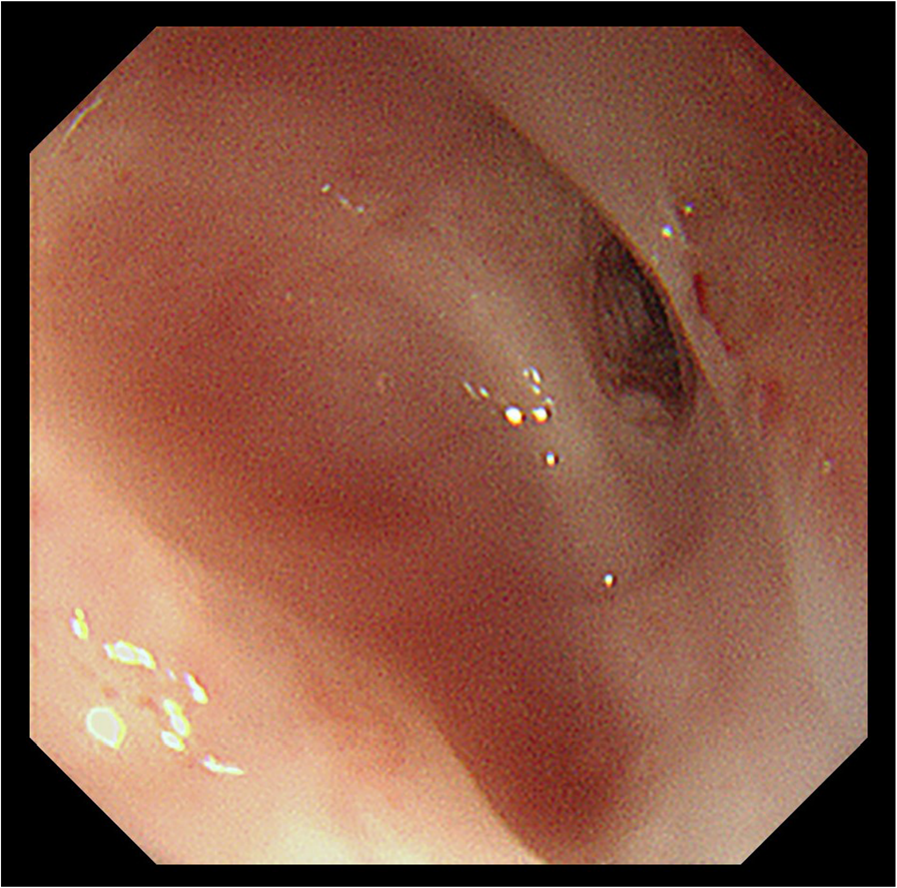
Endoscopic finding of 7 months after the initial operation. The stricture of the residual cervical esophagus

**Fig. 6:**
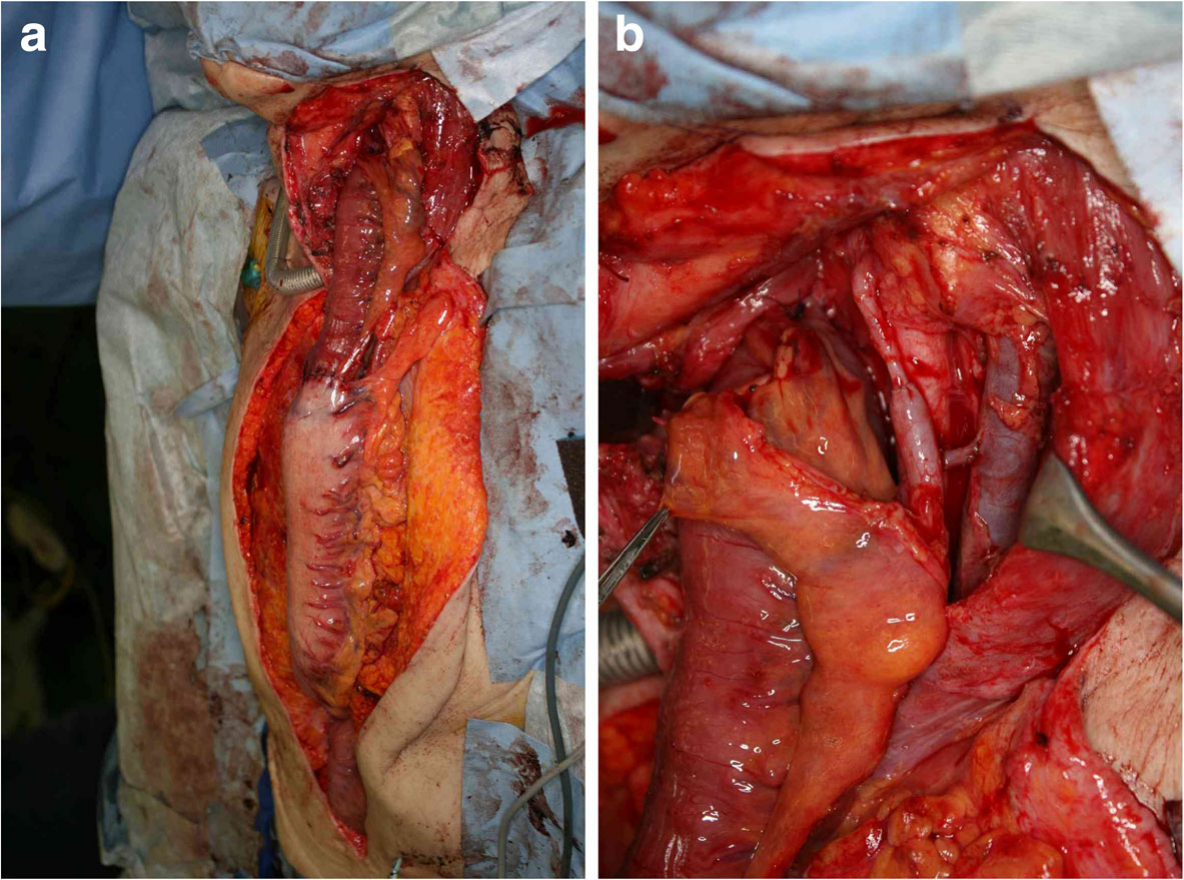
Findings of the reconstruction operation. **a** Reconstruction via an anterior thoracic route with free jejunum interposition and gastric tube. **b** Vascular anastomosis of the feeding artery and drainage vein of free jejunum. End-to-end anastomosis of the jejunal artery and left superior thyroid artery. End-to-side anastomosis of the jejunal vein and left internal jugular vein

The patient recovered generally well, except that the severe stricture of the residual cervical esophagus had developed a month after the operation. The stricture was endoscopically dilated repeatedly. However, the dilation was not successful enough, and 7 months after the initial operation, endoscopic balloon dilation (EBD) was required once a week (Fig. 5). Nine months from the first operation, resection of the stricture was performed using cervical esophagectomy accompanied by tracheotomy. Reconstruction was performed with free jejunum interposition between the hypopharynx and gastric tube through the anterior thoracic route (Fig. 6a). We used the superior thyroid artery and internal jugular vein for feeding artery and drainage vein of the free jejunum (Fig. 6b). No postoperative complications occurred, and she was transferred to rehabilitation therapy to continue swallowing rehabilitation on the 37th day after the operation. Her enterostomy tube was removed 3 months after the operation; she is now eating well and doing fine after all operations and therapy.

## Discussion

For the management of AEN in the acute phase, treatment is aimed at restoring hemodynamic stability and correcting underlying conditions and includes *nil-per-os* restriction, blood transfusions, and high-dose PPI therapy [10]. Insertion of a nasogastric tube is not recommended because of the risk of esophageal perforation [11]. Surgical intervention is reserved for perforated esophagus with resultant mediastinitis and abscess formation [11].

The most serious complication of AEN is perforation, which should be suspected in rapidly decompensating patients. Esophageal perforation may lead to rapid clinical deterioration from mediastinitis, mediastinal abscess formation, empyema, and generalized sepsis [11]. An immediate investigation is important if a perforation is suspected from clinical findings. Surgical intervention, such as esophagectomy, is necessary as soon as a perforation has been detected. Primary closure of the perforated esophageal tissue or primary reconstruction should not be attempted because saving the life of the patient should take priority at this point [11]. Six patients with perforated AEN undergone surgical intervention on literature review [2, 12]. One case involved a thoracoscopic approach placing an intrathoracic flushing system drain near the perforation [12]. However, in this case, the esophagus appeared normal from the outside, suggesting that there was no transmural necrosis of the esophagus [12]. The other cases involved an open approach. Except in one case, there were situations thought to be difficult to manage with the thoracoscopic approach, such as external compression due to ruptured thoracic aorta, gastric volvulus, and left main bronchus perforation [2].

In the present case, the patient was in shock at admission. However, signs of perforation were not observed, and the patient gradually stabilized with conservative therapy. Perforation and mediastinitis were suspected from the high fever and the increase of the right pleural effusion from the chest X-ray findings. A CT scan, which detected the free air in the mediastinum, was performed with proper timing and led to immediate surgical intervention. The underlying condition of this patient was diabetic ketoacidosis and not complicated with difficult situations; to avoid additional stress from surgical invasion, we selected a thoracoscopic approach that was useful for confirming the diagnosis and subsequent therapeutic operation.

Esophageal stricture is a complication in the chronic phase of AEN. When reviewing literature, 20 of 164 patients progressed to esophageal stricture within 3 weeks to 2 months after the onset of AEN [8, 12–20]. The initial treatment in most AEN-induced stricture cases was endoscopic balloon dilation (EBD). One patient without EBD progressed to complete obstruction after 3 months and was admitted 1 year and 4 months after the occurrence of AEN because of her strong desire for oral intake [13]. Bypass operation was performed after 8 months of rehabilitation to improve the tolerance to the operation [13]. Five patients received surgical treatment for stricture refractory to EBD; three patients received esophagectomies, and two patients received bypass operations [7–9]. Except for the previous case, the interval from esophageal stricture to surgical intervention was within 3 to 7 months [7–9]. Shichinohe et al. suggest cases that exhibit long-range esophageal stenosis and refractory to multiple EBD for several months as the indication of surgical intervention [8]. Esophagectomy, rather than bypass surgery, is the preferred procedure from the perspective of the risk of carcinogenesis due to chronic inflammation [13]. However, the underlying condition of AEN patients is generally poor, and bypass operation may be an alternative to reconstruction.

In the present patient, stricture was refractory to multiple EBD and was decided to be a surgical indication in consideration of frequent dilation. The entire esophagus was affected by inflammation and a stricture developed in the residual cervical esophagus. Therefore, the cervical esophagus needed to be removed, and anastomosis had to be performed at the hypopharynx with appropriate grafts, such as gastric tubes with free jejunal grafts as needed.

## Conclusion

Esophagus perforation and mediastinitis are lethal complications of AEN, and early detection is required to avoid life-threatening situations. Thoracoscopic drainage and subtotal esophagectomy should be attempted to manage these situations. If the cervical esophagus is also affected and exhibits stricture, this area must be removed, and an anastomosis needs to be performed at the hypopharynx using the appropriate graft.

## Abbreviations

AEN: Acute esophageal necrosis
EBD: Endoscopic balloon dilation
PPI: Proton pump inhibitor
